# Supplementary material for: Thrombocytopenia and increased risk of adverse outcome in COVID-19 patients
Source: PeerJ. 2022 Jun 30;10:e13608. doi: 10.7717/peerj.13608 (PMC9250762; doi:10.7717/peerj.13608)

**Supplementary appendix**

Supplement to: The association between thrombocytopenia and systemic disorders in COVID-19 patients

**S1 Table. Basic information of the COVID-19 patients in the study.**

| **Characteristics** | **Total** | **Thrombocytopenia** | **Non-thrombocytopenia** | **P value** |
| --- | --- | --- | --- | --- |
|  | **(N=2209)** | **(n=127)** | **(n=2082)** |  |
| **Age, years (median, IQR)** | 61 (47-69) | 67 (56-75) | 60 (46-69) | <0.001 |
| ≤45 | 514 (23.3) | 13 (10.2) | 501 (24.1) | <0.001 |
| 45-60 | 585 (26.5) | 29 (22.8) | 556 (26.7) |  |
| >60 | 1110 (50.2) | 85 (66.9) | 1025 (49.2) |  |
| **Sex, male, n(%)** | 1078 (48.8) | 93 (73.2) | 985 (47.3) | <0.001 |
| **Days from disease onset to clinic visit, median (IQR)** | |  |  |  |
|  | 9 (5-14) | 8 (5-12) | 9 (5-14) | 0.142 |
| **Length of stay, days, median (IQR)** | |  |  |  |
|  | 17 (12-24) | 14 (7-21) | 18 (12-24) | <0.001 |
| **Any comorbidity, n(%)** | 881 (39.9) | 68 (53.5) | 813 (39) | 0.001 |
| Hypertension | 699 (31.6) | 55 (43.3) | 644 (30.9) | 0.004 |
| Diabetes | 287 (13) | 23 (18.1) | 264 (12.7) | 0.077 |
| Coronary heart disease | 173 (7.8) | 14 (11) | 159 (7.6) | 0.168 |
| Cerebral infarction | 100 (4.5) | 8 (6.3) | 92 (4.4) | 0.322 |
| **Symptoms** |  |  |  |  |
| Fever | 1678 (76) | 107 (84.3) | 1571 (75.5) | 0.024 |
| Cough | 1438 (65.1) | 80 (63) | 1358 (65.2) | 0.608 |
| Fatigue | 705 (31.9) | 54 (42.5) | 651 (31.3) | 0.008 |
| Anhelation | 515 (23.3) | 37 (29.1) | 478 (23) | 0.11 |
| Anorexia | 510 (23.1) | 45 (35.4) | 465 (22.3) | 0.001 |
| Chest tightness | 493 (22.3) | 33 (26) | 460 (22.1) | 0.307 |
| Diarrhea | 242 (11) | 5 (3.9) | 237 (11.4) | 0.009 |
| Nausea | 132 (6) | 10 (7.9) | 122 (5.9) | 0.353 |
| Headache | 66 (3) | 3 (2.4) | 63 (3) | 0.874 |

IQR, interquartile range. COVID-19, coronavirus disease 2019.

**S2 Table.** **Multivariate analysis on the associations between thrombocytopenia and** **adverse outcome for COVID-19 patients in the study.**

| **Parameter** | **Thrombocytopenia** | **Non-thrombocytopenia** | **Multivariate** | |
| --- | --- | --- | --- | --- |
|  |  |  | OR (95% CI) | P value |
| ARDS |  |  |  |  |
| No | 34 | 510 | Reference |  |
| Yes | 4 | 16 | 3.05 (0.9-10.29) | 0.072 |
| Respiratory failure |  |  |  |  |
| No | 25 | 521 | Reference |  |
| Yes | 18 | 95 | 2.53 (1.25-5.14) | 0.010 |
| ICU entrance |  |  |  |  |
| No | 32 | 570 | Reference |  |
| Yes | 11 | 46 | 2.77 (1.38-5.59) | 0.004 |
| Acute heart injury |  |  |  |  |
| No | 39 | 594 | Reference |  |
| Yes | 4 | 22 | 1.64 (0.52-5.22) | 0.400 |
| Acute kidney injury |  |  |  |  |
| No | 41 | 601 | Reference |  |
| Yes | 2 | 15 | 1.25 (0.27-5.91) | 0.776 |
| Septic shock |  |  |  |  |
| No | 39 | 605 | Reference |  |
| Yes | 4 | 11 | 3.71 (0.99-13.87) | 0.051 |
| DIC |  |  |  |  |
| No | 60 | 1411 | Reference |  |
| Yes | 42 | 109 | 7.17 (4.56-11.28) | <0.001 |

The association between thrombocytopenia and adverse outcome for COVID-19 were calculated by the multivariate logistic regression models adjusted for age, sex, delay from symptom onset to admission and comorbidities. COVID-19, coronavirus disease 2019; ARDS, acute respiratory distress syndrome; DIC, disseminated intravascular coagulation; ICU, intensive care unit.

**S3Table. Comparison of laboratory indicators between the groups of patients stratified by platelet counts on admission at three stages.**

| **Parameter** | **P value at 1-10 days** |  | **P value at 11-20 days** |  | **P value at 21-30 days** |
| --- | --- | --- | --- | --- | --- |
| Neutrophil (%) | 0.762 |  | 0.239 |  | <0.001 |
| Neutrophil (×10^9^/L) | 0.435 |  | 0.015 |  | 0.046 |
| Lymphocyte (%) | 0.517 |  | 0.012 |  | 0.002 |
| Lymphocyte (×10^9^/L) | 0.921 |  | 0.256 |  | 0.979 |
| Monocyte (%) | 0.023 |  | 0.005 |  | 0.001 |
| Monocyte (×10^9^/L) | 0.004 |  | 0.008 |  | <0.001 |
| Eosinophil (%) | <0.001 |  | 0.869 |  | <0.001 |
| Eosinophil (×10^9^/L) | <0.001 |  | 0.467 |  | <0.001 |
| Basophil (%) | 0.026 |  | 0.050 |  | 0.024 |
| Basophil (×10^9^/L) | 0.178 |  | 0.839 |  | 0.002 |
| Mean platelet volume (fL) | <0.001 |  | 0.008 |  | 0.597 |
| Thrombocytocrit (%) | <0.001 |  | <0.001 |  | <0.001 |
| Platelet distribution width (fL) | 0.103 |  | 0.099 |  | 0.324 |
| Prothrombin time (s) | 0.021 |  | 0.032 |  | 0.030 |
| Activated partial thromboplastin time (s) | 0.005 |  | 0.006 |  | 0.583 |
| Thrombin time (s) | 0.077 |  | 0.001 |  | 0.831 |
| Fibrinogen (g/L) | <0.001 |  | 0.002 |  | 0.442 |
| International normanlizationg ratio | 0.157 |  | 0.082 |  | 0.232 |
| D-dimer (μg/L) | 0.508 |  | 0.097 |  | 0.425 |
| Fibrin degradation products (mg/L) | 0.039 |  | 0.004 |  | 0.077 |
| IL-6 (pg/mL) | 0.045 |  | 0.027 |  | 0.261 |
| TNF-α (pg/mL) | 0.004 |  | 0.302 |  | 0.673 |
| C-reactive protein (mg/L) | 0.188 |  | 0.036 |  | 0.372 |
| Procalcitonin (ng/mL) | 0.015 |  | 0.092 |  | 0.243 |
| Aspartate aminotransferase (U/L) | 0.077 |  | 0.085 |  | 0.205 |
| Total protein (g/L) | 0.407 |  | 0.009 |  | 0.062 |
| Albumin (g/L) | 0.243 |  | 0.005 |  | 0.008 |
| Urea (mmol/L) | 0.912 |  | 0.001 |  | 0.023 |
| Lactate dehydrogenase (U/L) | 0.032 |  | <0.001 |  | 0.080 |
| Creatine kinase-MB (U/L) | 0.699 |  | 0.293 |  | 0.022 |

P values were calculated by the generalized estimated equation adjusted for age, sex, delay from symptom onset to admission and comorbidities.

**S4 Table. Basic information of the COVID-19 patients with and without delayed-phase thrombocytopenia.**

| **Characteristics** | **Total** | **Patients with early development of thrombocytopenia** | **Patients with late development of thrombocytopenia** | **P value** |
| --- | --- | --- | --- | --- |
|  | **(N=245)** | **(n=184)** | **(n=61)** |  |
| **Age, years (median, IQR)** | 66 (54-75) | 65 (53.5-75.25) | 69 (56-75) | 0.389 |
| ≤45 | 33 (13.5) | 25 (13.6) | 8 (13.1) | 0.458 |
| 45-60 | 58 (23.7) | 47 (25.5) | 11 (18) |  |
| >60 | 154 (62.9) | 112 (60.9) | 42 (68.9) |  |
| **Sex, male, n (%)** | 150 (61.2) | 116 (63) | 34 (55.7) | 0.310 |
| **Days from disease onset to clinic visit, median (IQR)** | | |  |  |
|  | 7 (4-9) | 6 (3-8) | 9 (7-10) | <0.001 |
| **Length of stay, days, median (IQR)** | | |  |  |
|  | 17 (10-24) | 16 (9-23) | 21 (14-30) | 0.001 |
| **Any comorbidity, n (%)** | 124 (50.6) | 94 (51.1) | 30 (49.2) | 0.796 |
| Hypertension | 100 (40.8) | 73 (39.7) | 27 (44.3) | 0.527 |
| Diabetes | 44 (18) | 36 (19.6) | 8 (13.1) | 0.255 |
| Coronary heart disease | 31 (12.7) | 19 (10.3) | 12 (19.7) | 0.057 |
| Cerebral infarction | 19 (7.8) | 13 (7.1) | 6 (9.8) | 0.671 |
| **Symptoms, n (%)** |  |  |  |  |
| Fever | 208 (84.9) | 155 (84.2) | 53 (86.9) | 0.617 |
| Cough | 159 (64.9) | 114 (62) | 45 (73.8) | 0.094 |
| Fatigue | 125 (51) | 95 (51.6) | 30 (49.2) | 0.740 |
| Chest tightness | 110 (44.9) | 85 (46.2) | 25 (41) | 0.478 |
| Anorexia | 81 (33.1) | 59 (32.1) | 22 (36.1) | 0.565 |
| Anhelation | 61 (24.9) | 49 (26.6) | 12 (19.7) | 0.276 |
| Diarrhea | 21 (8.6) | 17 (9.2) | 4 (6.6) | 0.517 |
| Nausea | 21 (8.6) | 13 (7.1) | 8 (13.1) | 0.144 |
| Headache | 7 (2.9) | 6 (3.3) | 1 (1.6) | 0.829 |

IQR, interquartile range. COVID-19, coronavirus disease 2019.

**S1 Figure. Dynamic profile of platelet counts in thrombocytopenia patients with COVID-19 in different groups based on the PSM database.** A, platelet counts in survival and fatal group; B, platelet counts in non-hypertension and hypertension group; C, platelet counts in non-diabetes and diabetes group; D, platelet counts in non-cardiovascular disease and cardiovascular disease group; E, platelet counts in non-cerebral infarction and cerebral infarction group. The p value was calculated by generalized estimating equation regression model.


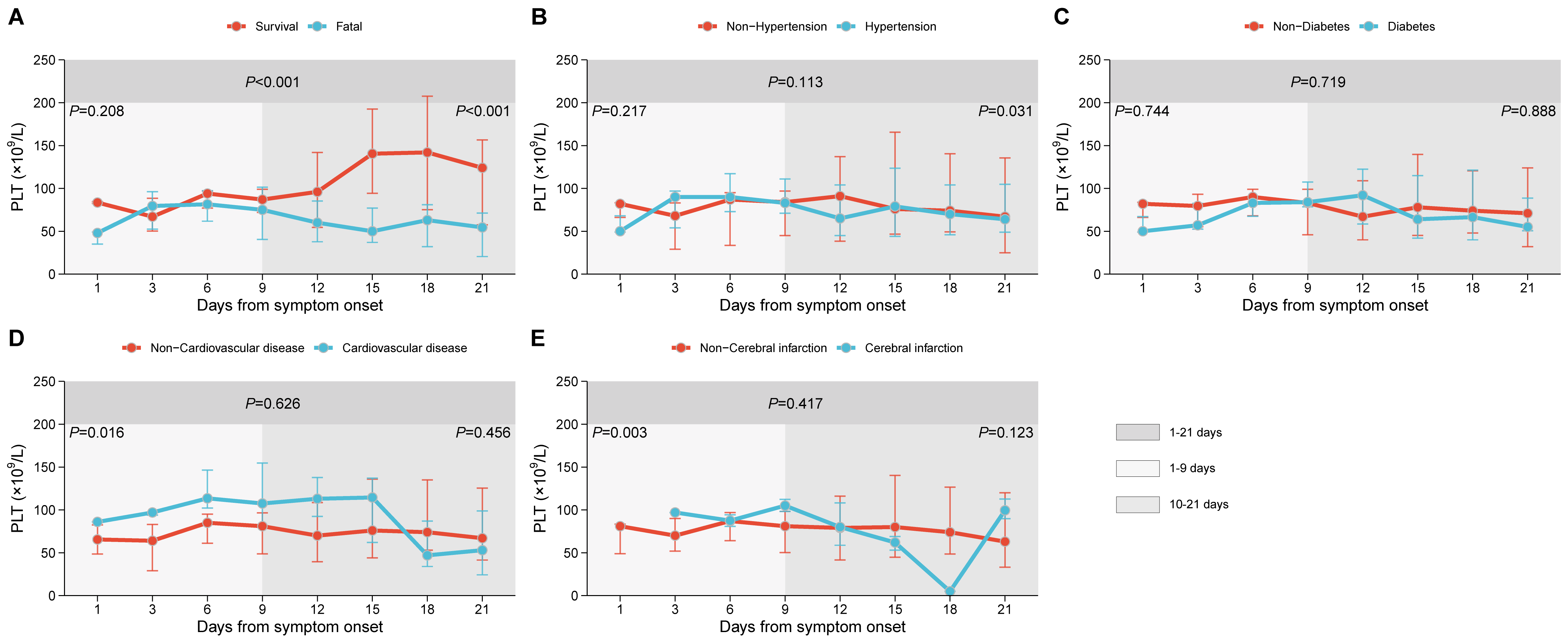


**S2 Figure. Laboratory indicators of patients with early and late development of thrombocytopenia.** A, neutrophil count; B, lymphocyte count; C, thrombocytocrit; D, international normalization ratio (INR); E, C reaction protein (CRP); F, procalcitonin. Dots present the values for individual detection, and the lines and error bars indicate the median and interquartile range, respectively. Dotted line indicates the lower limit and upper limit of each laboratory indicator. *P < 0.05 using a generalized estimating equation regression model adjusted for age, sex, delay from disease onset to hospital admission and comorbidities. The markers in comparisons without significant differences were not shown.


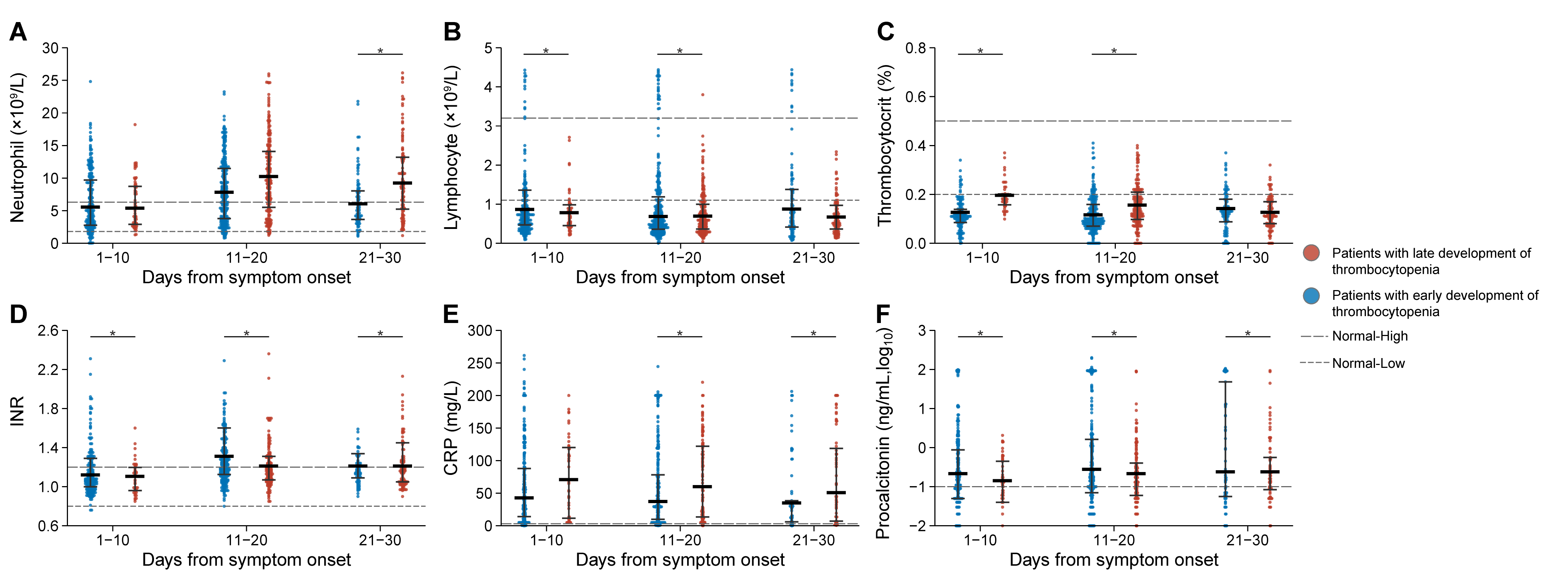


**S3 Figure. The dynamic profile of platelet counts and 60-day Kaplan-Meier survival curves of COVID-19 patients with early and late development of thrombocytopenia.** A, dynamic profile of platelet count was plotted in the two groups of patients with early and late development of thrombocytopenia. The median and interquartile range were shown. B, Survival curves on probability of survival from the patients with early and late development of thrombocytopenia and compared by log-rank test.


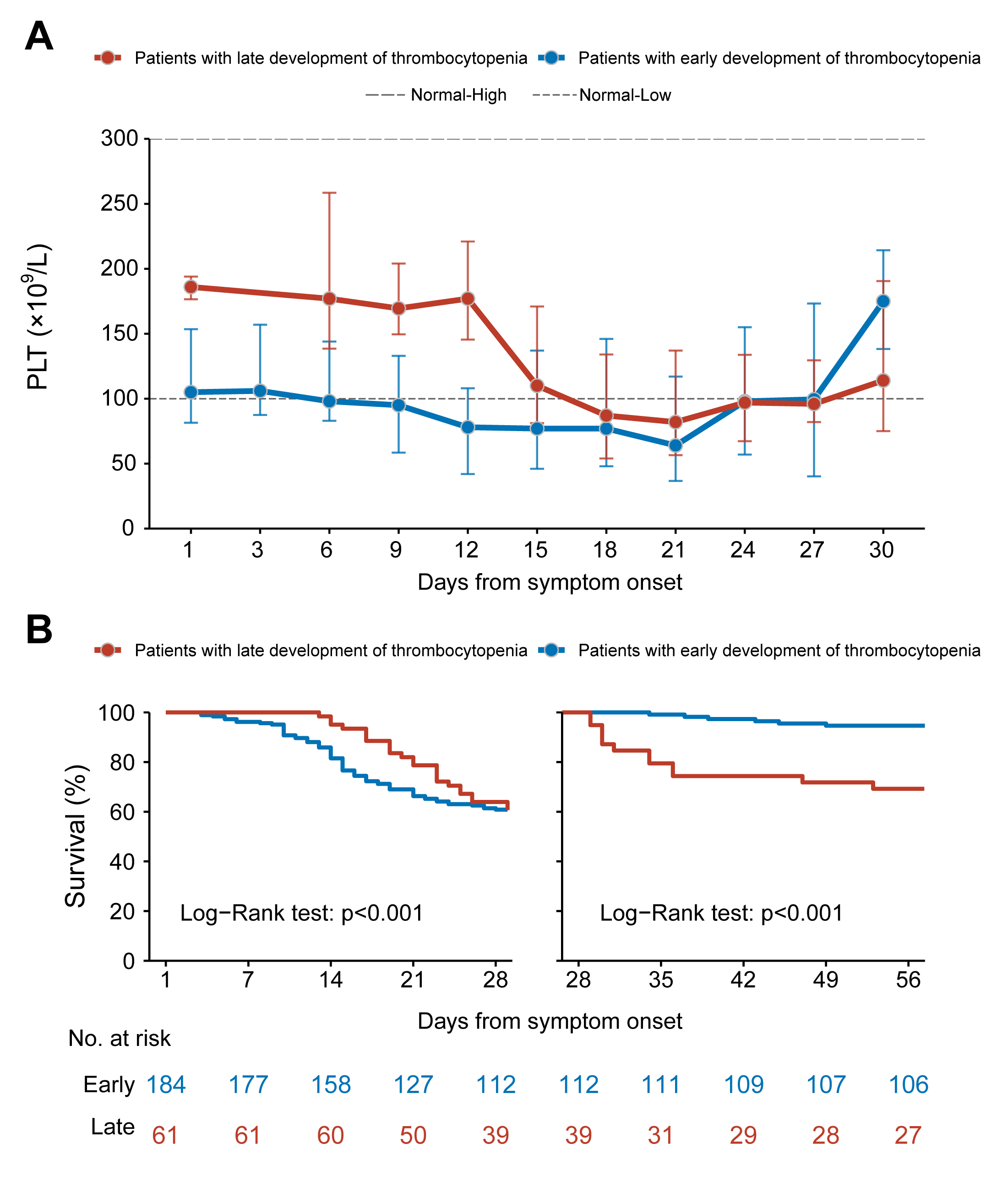

Supplement: Supplemental Information 1 [file peerj-10-13608-s001.docx]
